# Cooperative rescue of a juvenile capuchin (*Cebus imitator*) from a *Boa constrictor*

**DOI:** 10.1038/s41598-020-73476-4

**Published:** 2020-10-08

**Authors:** Katharine M. Jack, Michaela R. Brown, Margaret S. Buehler, Saul Cheves Hernadez, Nuria Ferrero Marín, Nelle K. Kulick, Sophie E. Lieber

**Affiliations:** 1 Department of Anthropology, Tulane University, 6823 St. Charles Avenue, New Orleans, LA 70118 USA; 2 Sector Santa Rosa, Área de Conservación Guanacaste, Liberia, Costa Rica

**Keywords:** Zoology, Ecology, Behavioural ecology, Community ecology, Tropical ecology, Evolution, Anthropology, Evolutionary theory, Sexual selection, Social evolution

## Abstract

The threat of predation by snakes is considered to have played a significant role in the evolution of primate sensory systems and behavior. However, we know relatively little about individual and group responses given the rarity of observed predation events. Here we report an observed (filmed) predation attempt by an adult *Boa constrictor* (~ 2 m) on a juvenile white-faced capuchin (*Cebus imitator*) in the Sector Santa Rosa of the Área de Conservación Guanacaste, Costa Rica. The snake caught the juvenile monkey on the ground during a terrestrial play session. When the victim screamed, the alpha male, alpha female, and another adult female ran to the scene, physically attacked the snake (with bites and hits), and pulled the victim to safety. Most group members participated in the vocal mobbing of the snake both during and after the attack. Based on the outcomes of this predation attempt and published reports of other *B. constrictor* attacks on primates, the coordinated efforts of ≥ 2 group members is needed for a successful rescue. This observation adds to our growing knowledge of cooperative group behavior and its importance in predator defense.

## Introduction

Predation is considered a major selective force leading to group living in many animal species^1^, including primates ^2–4^, though documented predation attempts are rare. Isbell^5^ suggested that the threat of predation specifically by snakes played a crucial role in shaping primate behavior and sensory systems. Given the wide geographic overlap between primates and snakes, it is not surprising that primates also developed an array of anti-predator behaviors that increase snake detection, such as vigilance behaviors, and deter predation, such as predator-specific alarm calls^6^ and predator mobbing^7^. This latter tactic involves animals attacking a predator vocally (repeated alarm calls) and/or physically (e.g., hitting, biting, or throwing items at a predator) rather than running away^7^. In many species, mobbing behavior is cooperative (involving multiple group members), alerts other individuals to the location of predators, and can drive predators away and discourage future hunting in the area^8^. Though effective in many cases, predator mobbing, particularly with non-ambush predators, does have potential costs as it can result in the injury or death of individual participants^9^. Mobbing ambush predators, such as constricting snakes, is a relatively common behavior for primates as it is much less risky and the hunting success of ambush predators is greatly reduced once they are detected^7^. Here we report on an observed predation attempt by a *Boa constrictor* on a juvenile white-faced capuchin (*Cebus imitator*) and the subsequent mobbing and rescue by group members.

Despite the assumed threat snakes pose on primates, there are few published observations of predation events. Based on these limited reports, the consequences of snake predation events are usually fatal. Of course, the efficacy of defensive tactics will differ when individuals are attacked by venomous or constricting snakes. Attempting to rescue a group member is more dangerous if a snake is venomous, as it could easily envenomate and kill multiple individuals and there is little an individual and/or their group members can do to save an envenomated individual. Four attacks on wild primates by venomous snakes have been observed and published, with the victim dying in all instances^10–12^. However, in the case of attacks by constricting snakes, escapes can occur with the aid of group members, as the risk of engaging a constricting snake is much lower once the snake begins coiling around the victim. Indeed, of the 16 documented predation events on primates by constricting snakes (14 Boidae, 2 Pythonidae), seven reported interventions involving one or more group members physically interacting with the predator (“rescue attempts”) (Table 1). Five of these interventions resulted in the successful release and survival of the victim. Note that four additional predation attempts were documented, but we are excluding these from analyses as the observers did not witness the initial attack and response of group members or humans intervened in the attack to save the primate victim. To our knowledge, these are the only published accounts of predation attempts on primates by snakes.

**Table 1:** Description of published constricting snake attacks on non-human primates.

Primate	Snake	Victim	Group response	Source
**Platyrrhines**				
*Alouatta puruensis*	*Boa constrictor*	Adult female (died, failed rescue)	Calls from many group members and attack from 1 female	^34^
*Callicebus discolor*	*Boa constrictor*	Adult (died)	Calls only	^36^
*Cebus imitator*	*Boa constrictor*	Small juvenile (died)	Calls from many group members and stick dropping, no physical attack	^37^
*Cebus imitator*	*Boa constrictor*	Small juvenile **(rescued)**	Calls from all group members and attack from 3 individuals	^14^
*Cebus imitator*	*Boa constrictor*	Juvenile **(rescued)**	Calls from all group members and physical attack from 3 to 4 group members	This paper
*Callithrix penicillata*	*Boa constrictor*	2 juveniles (died) failed rescue	Calls from some members and physical attack from 2 individuals, though the 2nd only participated for a few seconds	^35^
*Chiropotes satanas utahicki*	*Boa constrictor*	Adult female (died)	Calls only	^38^
*Saguinus mystax*	*Boa constrictor*	Subadult male **(rescued)**	Calls, mobbing, and physical attack from 2 adults	^51^
*Saguinus mystax*	*Eunectes murinus*	Adult female (died)	Calls only	^39^
**Other primates**				
*Propithecus coquereli*	*Acrantophis madagascariensis*	Adult female **(rescued)**	Calls and physical attack from > 3 adults (up to 8 but exact number is unclear)	^52^
*Microcebus murinus*	*Sanzina malagascariensis*	Adult male **(rescued)**	Calls from some group members and physical attack from 3 individuals	^53^
*Tarsius spectrum*	*Python reticulatus*	Unknown (died, failed rescue)	Calls from some group members and 1 individual bit the snake once	^33^
**Other constricting snake predations: (excluded from discussion due to lack of data or human intervention)**				
*Saimiri sciureus*	*Corallus hortulanus*	Adult female (died)	Unknown. Predation observed as snake was swallowing	^54^
*Propithecus verreauxis coquereli*	*Acrantophis madagascariensis*	Adult female (rescued by humans)	Calls only. Individual freed by human observer. Author suggested the victim would have died without intervention	^40^
*Nycticebus coucang*	*Python reticulatus*	Adult male (died)	Predation was assumed (not observed). Python was found with an ingested radio collar	^55^
*Hapalemur griseus griseus*	*Sanzinia madascariensis*	Adult (died)	Unknown. Predation was observed as snake was swallowing	^56^

**Figure 1:**
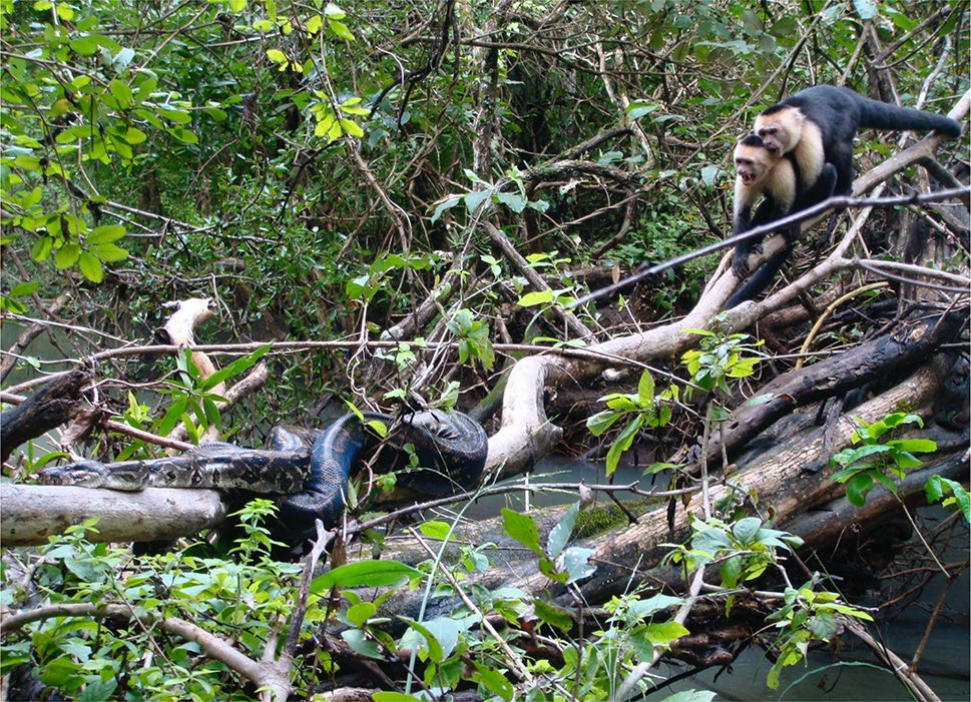
An alpha male and a subadult male white-faced capuchin cooperatively threaten a *Boa constrictor* in the SSR, Costa Rica. Photo by Jeffrey A. Rinderknecht, courtesy of Valerie Schoof.

Here, we add to these limited observations by reporting an attempted predation, and successful rescue, of a juvenile white-faced capuchin (*Cebus imitator*) by a *Boa constrictor* in the Sector Santa Rosa (SSR) of the Área de Conservación Guanacaste, Costa Rica. Our team was filming a terrestrial play session prior to the predation attempt, thereby enabling us to capture the details of the group response to the attack on film. The capuchins at SSR encounter snakes at a rate of 2.85/100 h of observation and cooperative mobbing of snakes is common^13^. Usually, several group members will cooperate in the mobbing by directing loud alarm calls and/or threat vocalizations towards the predator while engaging in threatening facial and branch shaking displays^14–17^. However, they do not usually come into direct contact with snakes and maintain a safe distance while threatening the predator (Fig. 1). Given the potential costs of directly engaging with a deadly snake^7^, and the rarity with which this type of behavior has been observed and/or reported, additional observations are critical for cross-species comparisons on variation in individual and group responses to predation. Such data will collectively advance our understanding of snake predation pressures and their selective role in the evolution of primate sociality and cooperative behavior.

## Methods

The SSR white-faced capuchins have been under intensive investigation since 1983, and members of multiple study groups are individually known and habituated^18^. The capuchins in SSR reside in groups ranging from 5 to 35 members and include multiple related females, multiple immigrant males, and their offspring^18^. SSR comprises approximately 100 km^2^ of deciduous tropical dry forest located 30 km south of the Nicaraguan border. SSR experiences distinct wet and dry seasons, with nearly all of the annual rainfall (avg. 1,792 mm) occurring in the wet season between mid-May and mid-November^18^. The predation attempt reported here took place on July 17, 2019, during the yearly *veranillo,* the characteristic dry period that occurs for several weeks during each wet season in July and/or August^19^. Our observations were made on the *Los Valles* (LV) group, which has been the focus of intensive research from 1991-present. At the time of the observation, group size was 25, including 4 adult males (≥ 10 years), 8 adult females (≥ 6 years), 4 large juveniles (4–6 years), 4 small juveniles (1–2 years), and 5 infants (≤ 1 year). White-faced capuchins are largely arboreal, though they spend considerable time both foraging and playing on or near the ground^20^.

**Figure 2:**
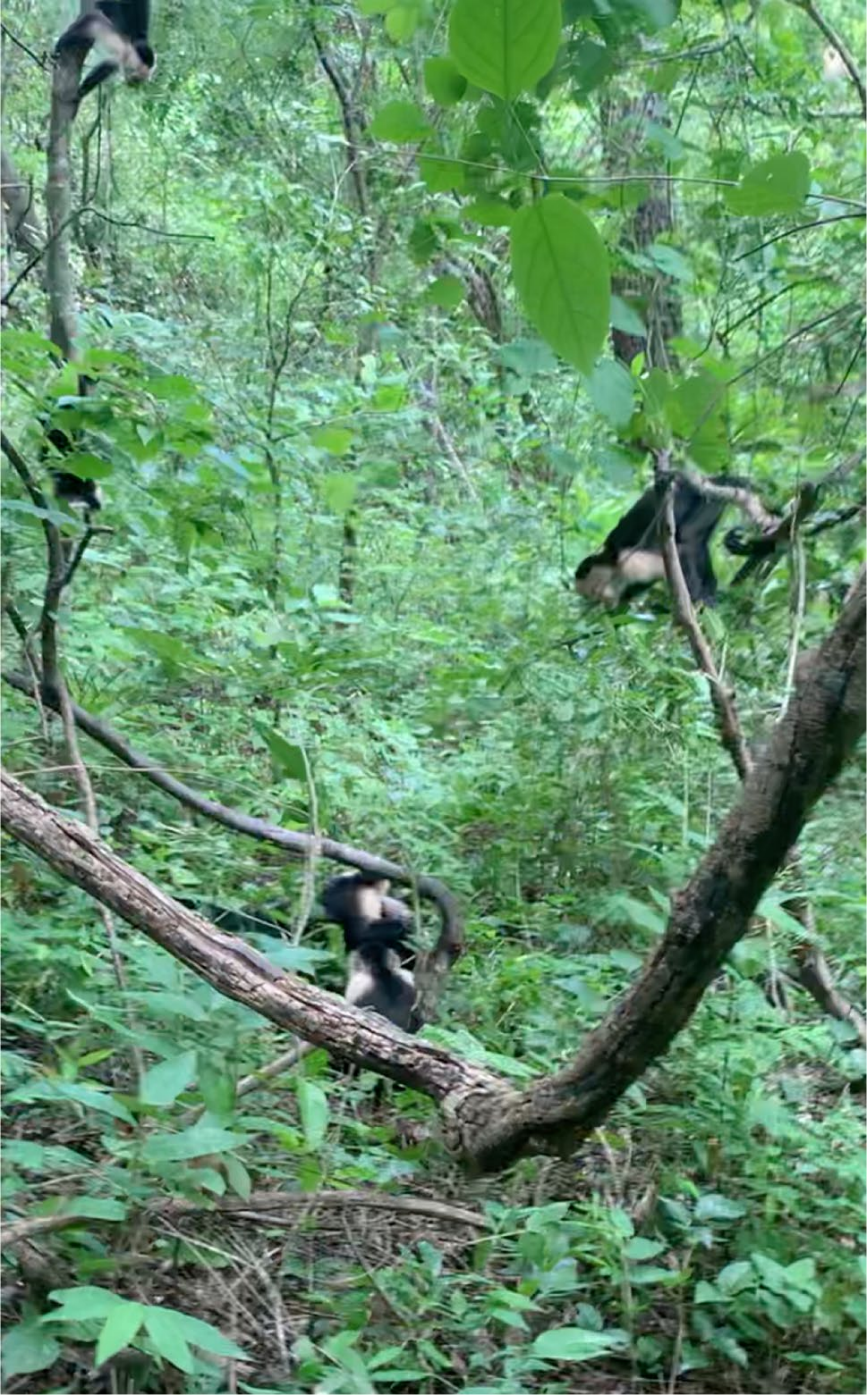
Screen shot from video of the rescue attempt (recorded by Sophie Lieber). Alpha male HP (center back) biting the boa, adult female TH (center front) actively pulling the victim from the snake, and three group members alarm calling at the scene. Note: many more were in attendance but out of the image frame.

The video was captured on an iPhone XS Max at 1080p HD at 30 frames per second (fps) (see https://drive.google.com/file/d/1sJeNZkgZ7iffzPHz_4Gsr0MrwtVPxbKl/view?usp=sharing). Video recording (by SEL) began 0:28 s prior to the first scream by the victim, marking the beginning of the attack. The video was analyzed using Adobe Premiere Rush to slow down the footage to 20% speed during the moment of attack, release, and aftermath. Additionally, we analyzed the video in QuickTime using the frame by frame function. Figure 2 is a screen shot from the video.

### Ethics statement

All research conducted in Santa Rosa and reported in this paper was authorized by the Costa Rican Ministry of the Environment, Energy and Technology (MINAET), and complied with protocols approved by Tulane’s Institutional Animal Care and Use Committee (IACUC).

## Results

Intermittent video recording of the group had been occurring for two hours prior to capturing the boa attack on video on July 17, 2019. The entire duration of the *B. constrictor* (boa herein) attack on the victim was less than 20 s, measured from when the screams started (0:28:82 in the video) to when the victim was released (0:47:19). Information on the key participants in the predation attempt and rescue are presented in Table 2.

**Table 2:** Key participants involved in the rescue.

Name code	Age-sex (age years)	Rank	Kinship
PW	Adult male (13.5)	Subordinate	Immigrant
HP	Adult male (10)	Alpha	Immigrant
SS (with 3 month old infant on her back)	Adult female (23)	Alpha	Mother of TH and OR
TH	Adult female (11)	Subordinate	Daughter of SS
OR (70% certainty)	Adult female (14)	Subordinate	Daughter of SS
Victim	Large Juvenile (~ 6)	Immature	Possibilities include the niece of SS, son of OR/grandson of SS, or son of CR (female unrelated to other participants)

At 11:26 am, an unidentified large juvenile (~ 6 years) was caught by an approximately 2 m long boa while engaged in a terrestrial play session. The individual ID is based on the victim’s body size in comparison to the known monkeys involved in the rescue. We were unable to definitively ID the victim due to the fast paced action of the attack, the obscured view in the video, and the victim’s quick flight from the scene following its release from the snake. Here we provide a time-stamped account of the events. While we made every attempt to ID all of the individuals involved, even with reviewing the film frame by frame, we were unable to confirm all IDs due to the fast action of the event. We provide individual IDs only for those animals that we were able to distinguish with 100% certainty.

0:00:00–0:28:81 The majority of group juveniles and several adults were engaged in a play session that involved reciprocating chases, running along the ground, and jumping up and down between tree trunks, vines, and the ground.

0:28:82—Screams of the victim are heard and the camera is directed towards him/her. The victim’s screams can be heard throughout the duration of the attack.

0:29:88—First snake alarm call is sounded; adult male PW, who was sitting on the ground 1.5 m from the boa (separated by vines and green vegetation), jumped from the ground up to a branch about one meter above the snake and began to alarm call while facing the snake.

0:34:62—Alpha male (HP) runs along the ground up to the boa and immediately makes contact with the snake (possibly biting it).

0:35:85—Alpha female (SS), with her 3 month old infant clinging to her back, follows on the ground after HP and also approaches the snake and victim.

0:38:52—SS jumps up to the branch with PW and both continue alarm calling at the boa.

0:38:53—Adult female TH (11 year old daughter of SS) runs on the ground and joins HP and the boa (the boa is between the two monkeys). HP hits, scratches, and possibly bites the snake and TH also appears to bite the snake several times and pulls at the victim.

0:44:13—An unidentified adult female (possibly OR, the daughter of SS and sister of TH) with an infant on her back approaches the snake to the left of HP, alarm calling and possibly hitting the snake.

0:44:16—HP lunges forward and bites the boa. The unidentified adult female with the infant retreats up the tree behind HP.

0:45:65—HP holds the snake with both hands and bites it. Blood is visible on the snake after the bite. TH is actively pulling the victim from the snake (Fig. 2).

0:46:33—SS approaches the group on the ground, PW (still on the branches to the right of the snake) lunges down the tree closer to the snake.

0:47:25—SS joins the adult female in pulling the victim as HP releases his bite from the snake.

0:47:19—The victim’s screams cease as it escapes from the snake and rolls out, knocking over SS and her infant, and runs quickly from the scene.

0:47:61—HP removes his hands from the snake and retreats, jumping into the overhead tree and joining another group member in threatening the snake (vocal and facial threats).

0:48:17—TH pulls herself up to vine directly above her (and beside the snake). She sits and alarm calls and directs facial threats towards the snake.

0:51:36—SS jumps up to tree on the right, rejoining PW. They both alarm call and threaten the snake.

0:57:02—TH bends closer to the snake, emits one last facial threat and then runs along the vine away from the snake, and out of the video frame. At this time the snake begins moving away from the location of the attack.

Note, shown in the linked video: at 2:03:09—Adult female CH, three adult males (HP, HG, and BY) and an unidentified large juvenile actively threatening from a tree directly over the snake as they watch it move off and settle under some foliage and branches about 3 m away from the attack location. The snake remained motionless in this location, although it was still somewhat visible. The group remained in the area and continued to alarm call and direct threats and branch shaking towards the snake, well past the end of the video at 3:39 s. Approximately 20 min after filming ended, the group moved off to forage and our team followed the monkeys, leaving the snake behind.

Following the attack, several drops of blood were observed on the ground where the attack took place. Though we cannot know for certain that the blood was from the boa, there was blood visible on the snake following the bites by HP, who had fresh blood on his left hand and shoulder immediately following the event. HP showed no sign of injury after the rescue or in the days following the attack, thus we suspect that the blood on HP was from the snake. All other group members appeared to be unharmed, as none showed any injuries or issues with movement following the event.

## Discussion

Cooperative predator defense has been observed in a variety of animals, including small birds (*Hirundo rustica*^21^, *Malurus coronatus*^22^, *Manorina melanocephala*^23^), lobster (*Panulirus argus*^24^), giant otters (*Pteronura brasiliensis*^25^), and meerkats (*Suricata suricatta*^26^). However, cooperative rescues from predatory attacks, like the case we describe here, are rarely observed in wild vertebrates and to our knowledge, have only been reported for primates (Table 1; see also Ref.^27^), humpback whales (*Megaptera novaeagliae*^28^), banded mongoose (*Miunfos mungo*^29^), and possibly dolphins (Delphinidae^30^). These rescue behaviors are considered a special form of cooperation as they involve one or more individuals putting themselves at risk to aid another, with no guarantee that the outcome will be successful, and no direct gain for the rescuer(s)^31,32^.

Our observations described here clearly highlight the efficacy of cooperative predator mobbing to rescue a group member. Similar to Perry et al.^14^, we observed that with combined effort in the trees and on the ground, the group surrounded the snake and attacked from multiple angles while simultaneously pulling the victim loose. When taken together, these two observations provide strong evidence that rescue behaviors are part of the normal behavioral repertoire for *C. imitator*. This cooperation renders it difficult for the snake to defend itself and continue to hold the victim. In examining the published reports on constricting snake attacks on primates (Table 1), the coordinated attack by multiple group members to rescue a groupmate appears to be the key to a successful rescue. In all five successful snake predation rescues, the victim was released only after multiple group members (≥ 2) physically attacked the snake. In the three unsuccessful rescue attempts, the physical attack by group members was minimal, with one case involving only a single bite to the snake from one individual^33^, another case in which a single female hit the snake 4–10 times with her hands^34^, and in the final case a single individual jumped on the snake intermittently for 30 seconds and was joined by a second individual for the last few jumps^35^. In the four remaining cases of constricting snake predations for which sufficient observations were made, the group response was limited to alarm calls and the victim did not survive^36–39^. Similarly in the constricting snake predation attempt on a sifaka described by Burney^40^, group members only sounded alarm calls and observers were certain the victim would have perished without their intervention. While the number of recorded constricting snake attacks on wild primates remains low, collectively they illustrate that group mobbing behavior accompanied by physical aggression and the combined efforts from multiple individuals can be effective in saving group members.

Why individuals cooperate has been a central question in behavioral ecology, particularly in cases where they risk their own lives to help others. Indeed in 2005, the evolution of cooperative behavior was identified as one of the “top-25 big questions facing science over the next quarter century”^41^. Kinship certainly promotes cooperative behaviors (e.g.^42^), and in the boa rescue described by Perry et al.^14^, the victim’s mother was the main participant in the physical attack of the snake along with the group’s alpha male. Though we were unable to definitively identify the victim of the attack in our observation, we can limit the possibilities to just three large juvenile group members. Two of these three were closely related to the three adult females who most actively participated in the rescue.

While we cannot determine if kinship was a motivating factor for the adult females who rescued the victim, we know that none of the possible victims were related to the alpha male (he was not a member of the group during the period when any of the potential victims were sired). In the event described here, the alpha male was the most active participant in the defensive attack. It is doubtful that the victim would have escaped without his intervention, similar to the rescue reported by Perry et al.^14^. Within seconds of the victim’s screams, the alpha male ran to the scene and physically attacked the snake while a low-ranking, subordinate male (PW), who was right next to the boa when the attack occurred, only alarm called. Though anecdotal, these observations support the broader trend of alpha males as principal protectors of their groups. Alpha male capuchins are the most vigilant group members and the most active during interactions with predators and extragroup conspecifics^43,44^. They also maintain testosterone levels that are significantly higher than subordinate males^45–47^. These extreme concentrations are not necessary for reproductive function, as subordinate males with lower concentrations are capable of siring offspring^48,49^. These high levels of testosterone likely enable alpha male capuchins to remain alert and quickly to respond to threats^55,56^, which in this case the alpha male did even prior to siring offspring in the group.

Observations of group responses to predation events, though rare, clearly support the hypothesis that predation has been a strong selective force driving sociality in primates (e.g.^50^). While Isbell^5^ argued that the coevolution of snakes and primates have strongly influenced the evolution of primate visual systems, observations of cooperative group rescues of victims from constricting snakes further supports the strong role these predators have had in shaping primate behavior and sociality. Given that such cooperative rescues have now been reported for platyrrhines, tarsiers, and strepsirrhines but not in cercopithecines or hominoids, the large bodied primates, indicates that the threat of constricting snakes may have been a particularly strong selective force in early primate evolution when primates were small bodied and, therefore, more susceptible to fall prey to constricting snakes.

## Data Availability

All data relevant to this publication have been presented or supplied in the pubished manuscript.
